# Heterogeneous Algorithm for Efficient-Path Detection and Congestion Avoidance for a Vehicular-Management System

**DOI:** 10.3390/s23125471

**Published:** 2023-06-09

**Authors:** Melaouene Noussaiba, Abdul Razaque, Romadi Rahal

**Affiliations:** 1 Team of Information Research and Indexing Documents, Texts and Multimedia, ENSIAS, Mohammad V University, Rabat BP 713, Morocco; noussaiba.melaouene@um5r.ac.ma (M.N.); rahal.romadi@ensias.um5.ac.ma (R.R.); 2 School of Computing, Gachon University South Korea, Seongnam-si 13120, Republic of Korea

**Keywords:** ant-colony optimization, pheromone termite, congestion avoidance, energy consumption, end-to-end delay

## Abstract

Finding reliable and efficient routes is a persistent problem in megacities. To address this problem, several algorithms have been proposed. However, there are still areas of research that require attention. Many traffic-related problems can be resolved with the help of smart cities that incorporate the Internet of Vehicles (IoV). On the other hand, due to rapid increases in the population and automobiles, traffic congestion has become a serious concern. This paper presents a heterogeneous algorithm called ant-colony optimization with pheromone termite (ACO-PT), which combines two state-of-the-art algorithms, pheromone termite (PT) and ant-colony optimization (ACO), to address efficient routing to improve energy efficiency, increase throughput, and shorten end-to-end latency. The ACO-PT algorithm seeks to provide an effective shortest path from a source to a destination for drivers in urban areas. Vehicle congestion is a severe issue in urban areas. To address this issue, a congestion-avoidance module is added to handle potential overcrowding. Automatic vehicle detection has also been a challenging issue in vehicle management. To address this issue, an automatic-vehicle-detection (AVD) module is employed with ACO-PT. The effectiveness of the proposed ACO-PT algorithm is demonstrated experimentally using network simulator-3 (NS-3) and Simulation of Urban Mobility (SUMO). Our proposed algorithm is compared with three cutting-edge algorithms. The results demonstrate that the proposed ACO-PT algorithm is superior to earlier algorithms in terms of energy usage, end-to-end delay, and throughput.

## 1. Introduction

Existing transportation systems are constantly under serious pressure as the number of vehicles and the need for mobility have enormously increased in densely populated and urban areas. Accordingly, finding effective and reliable routes has become a common logistics problem that has been researched for decades. The goal of a unique vehicle-routing problem is to find cost-effective routes for a large number of vehicles [1,2,3]. Meanwhile, new issues have arisen, especially in traffic factors such as low data rates, energy efficiency, congestion, and the fastest route [4,5,6]. This study focuses on seeking the most effective and the shortest route from a source point to a destination point in urban areas. Vehicle routing is an integrated programming problem that aims to determine the optimal set of routes for a set of vehicles. To address these issues, several efficient-routing protocols have been developed. A previous study [7] evolved an efficient linear-scheduling algorithm for multiple routes, which influences time delays caused by vehicles assigned to specific routes. The Multiple Access with Salvation Army (MASA) protocol was introduced to find the best route [8]. The proposed method combines several factors into a multi-weighted graph. These methods are intended for pedestrian tourists as well.

Other issues that arise due to routing concern the network lifetime and the energy consumption. For an efficient route, the ACO-based coverage-diversity framework was proposed [9,10]. This method addresses newly discovered customer requests (vehicles). Ant-colony optimization and hybrid butterfly algorithms were introduced for energy-efficient routing [11] to maximize the network lifetime. Along with this optimization, which makes the network scalable and improves performance, several swarm-intelligence-based approaches have been proposed by researchers to facilitate vehicle routing and to identify near-optimal routes in a reasonable amount of time. Most of the predominant strategies are based on ACO, particle-swarm optimization (PSO), artificial bee colony (ABC), flower-pollination cuckoo search (FPCS), and others [8,12,13,14].

These approaches address the issue of efficient routing with extension but overlook routing durability. Therefore, hybrid techniques have been proposed to address the optimized-routing problem. Hybridization includes the integration of two or more metaheuristic algorithms by mutually handling their limitations and balancing the trade-off among exploitation and exploration rates. Furthermore, hybrid algorithms integrate the best aspects of the chosen algorithms to produce the most effective algorithm. Because the ACO algorithm can initiate local searches only, the hybrid ant-colony-optimization–particle-swarm-optimization (ACO-PSO) method was introduced to increase the network lifetime. However, PSO provides global updates to ensure a reliable route [15]. A heterogeneous adaptive ant-colony-optimization algorithm was introduced to solve the traveling-salesman problem by providing an efficient route [16]. Ant beta and alpha values were adapted and mutated to address the problem of the worst-performing ant to find the best route. Two hybrid algorithms based on ACO were used to address an efficient neighborhood route [17].

To support wireless-sensor networks (WSNs), an efficient routing protocol was introduced [18]. With these premises, such an approach opens up possibilities to devise more effective and sustainable future mobility and transportation systems. Most existing approaches in the aforementioned contexts attempt to determine the best route but do not account for vehicle congestion or automatic detection, which is the motivation behind this work. They normally assume that these services are provided in a static and passive way, which explains the urgent need for a more efficient route that addresses traffic congestion and automatic vehicle detection.

**Figure 1:**
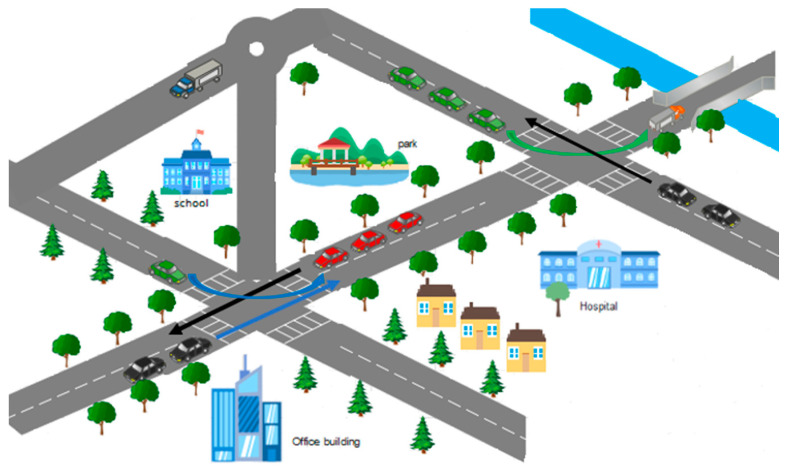
Efficient and reliable path-detection process.

In summary, the proposed automatic-vehicle-detection-module (AVDM) approach successfully provides an efficient and reliable route by avoiding congestion and automatically detecting vehicles, as shown in Figure 1.

The main contributions of this study can be summarized as follows:

- The heterogeneous algorithm is introduced, which combines the best aspects of two state-of-the-art algorithms (PT and ACO). ACO delivers an efficient and dependable approach using local and global search features. Furthermore, ACO incorporates real-time probabilistic and flexible properties. On the other hand, ACO has problems with convergence speed and accuracy when dealing with enormous amounts of data. To address the shortcomings of ACO, PT provides support for network scalability and vehicle mobility. The analytical model of PT is based on two separate parameters: packet-generation rate and pheromone sensitivity for single and multiple networks. Before delivering packets on each connection, the packet-generation rate minimizes congestion and pheromone sensitivity defines the link capacity. As a result, energy efficiency is improved.
- The novel congestion-avoidance model is intended to save fuel, minimize carbon emissions, and protect the environment. It is possible to achieve this by estimating the average speed of vehicle movement, which is affected by vehicle density. As a result, throughput is improved and energy usage is decreased.
- The automated-vehicle-detection module detects the presence and movement of vehicles in a given region by going through many phases (image preprocessing, contour detection, contour matching, and blob detection). As a consequence, the accuracy of automated detection is improved compared to other state-of-the-art methods.

The remainder of the paper is organized as follows: Section 2 presents the salient features of current approaches in related work. Section 3 provides the details of the proposed heterogeneous algorithm for efficient-path detection. Section 4 presents congestion-avoidance and vehicle-detection modules of ACO-PT for vehicles. Section 5 presents the experimental setup, including the results. Section 6 discusses the results, and finally, Section 7 concludes the entire paper with future work.

## 2. Related Work

Related literature emphasizes pertinent and contemporary approaches, as well as their positive and negative aspects. For optimum routing, the heterogeneous multidepot cooperative vehicle-routing problem (HMCVRP) is suggested [19]. The suggested technique outperformed the CPLEX solver, which employs the commonly used big-M transformation-based approach, in numerical tests. Further computer studies demonstrated the importance of depot locations and the implementation of an efficient cost-saving distribution system. The cuckoo-optimization algorithm was used by the authors of [20] to show energy-aware clustering-based routing (ECR) in wireless-sensor networks. The present study suggests an energy-conscious bunching-based guiding convention for wireless-sensor networks that can group the system and pick optimal cluster leaders using cuckoo-improvement computation. The suggested approach considers four criteria for choosing cluster leaders in the focused cuckoo computation, specifically, the remaining energy of the node, isolation from the base sink, inside-group separations, and amid-group separations. The suggested approach outperformed various calculations in MATLAB circumstances, such as low-energy adaptive-clustering-hierarchy (LEACH)-application explicit low-power directing, and low-energy adaptive-clustering-hierarchy energy prediction (LEACH-EP), with the parting-based edge as the core node dies by the large and packet rate of transmission in six different situations. The authors proposed an improved basis for the ACO algorithm based on fuzzy logic (ACO-FL) for vehicular ad hoc networks (VANETs) [21].

To test the efficacy and speed of the proposed protocol, the network simulator NS-2 was used. The simulation findings indicated that compared to current routing algorithms such as the ad hoc on-demand distance vector (AODV) and ACO, the proposed protocol achieved a reduced end-to-end delay and a greater data-packet delivery ratio. This proposed routing protocol establishes a hierarchical routing scheme in which nodes in the outer level forward data to nodes in the inner level [22]. Ant colonies are used to improve the routing route, ensuring that data travel on the least congested path feasible. Furthermore, when ant-colony optimization is used, certain cluster-head nodes may become overwhelmed with data forwarding, leading to early death due to an energy shortage. The quantity of data from the nearby cluster head is evaluated and transmitted based on their extra energy to surmount this anomaly. The authors concluded that the system saves more energy, prolonging the lifespan of the network, after comparing the energy-usage results of the proposed routing using ant-colony optimization (RACO) with those of other existing clustering algorithms.

A prediction approach was added to the current metaheuristic methodology [23]. A refined metaheuristic method is called ant-colony optimization. Instead of encouraging ants to seek higher pheromone densities, the suggested methodology forecasts pheromone characteristics based on middle pheromone densities. The intermediate stage’s variable pheromone quantities are simulated using the quantum approach. The stochastic differential equation and Ito integral are used to determine the pheromone features outlined below. The predicted pheromones function as parameters in subsequent ACO iterations. To verify the efficiency of the suggested architecture, shortest-route generation is provided for several large-scale networks. The latter is an extremely efficient metaheuristic paradigm that combines quantum-mechanism-based prediction with previously developed metaheuristics. For data transfer, the authors proposed AODV and ACO (AODV-ACO) [24]. The ant-colony-optimization method was used, which considers the initial coordinates of each node. The reactive-routing-protocol route had a high probability of creating network congestion and used substantial network bandwidth.

A route server that can handle all of the traffic in a city is supported by the introduction of the traffic-management paradigm [25]. By taking into consideration both current and projected levels of traffic congestion, the contribution balances traffic flows. The simulation research, which used actual data on traffic congestion in Valencia, Spain, showed how the suggested remedy would enhance traffic flow throughout an average day. According to experimental findings, the proposed traffic-prediction equation can significantly improve average travel speeds and travel times, two measures of reduced levels of congestion and increased traffic fluidity, when used in conjunction with frequent updates of traffic conditions on the route server. A novel approach for estimating statistical variables based on a unidirectional-road-network model was provided [26]. The suggested technique is more realistic, and it is a route-planning algorithm for periodically constrained search regions that generate virtual borders with a lower confidence level. A comprehensive experiment was carried out with the suggested method using the real road network in Zhengzhou. The suggested approach was compared to current methods. Based on the results, the suggested algorithm considerably increased search performance under the condition of the optimum path toward ensuring the best solution.

To avoid congestion, the time-dependent vehicle-routing problem with time windows (TDVRPTW) was presented [27]. A mathematical model for the TDVRPTW was developed by considering time-dependent vehicle speeds, travel time, capacity, customer demand, wait time, time window, service time, the influence of dynamic loads, and the effect of travel speed on vehicle carbon emissions. Furthermore, a congestion-avoidance strategy was provided to prevent peak-hour traffic congestion and temporal traffic congestion. The TDVRPTW properties of the model were used to develop an enhanced ant-colony algorithm with a congestion-avoiding strategy (IACACAA). Several computational tests proved the efficacy of the proposed methodologies. Congestion avoidance decreased vehicle fuel consumption and carbon emissions while protecting the environment. Energy-efficient message distribution (EEMD) based on a fog-assisted congestion-avoidance method for the IoV is described in [28]. In contrast to the majority of existing strategies, EEMD uses the advantages of fog computing to reduce communication costs and manage services. The status of every vehicle must be routinely updated to a fog server, either directly or through intermediary nodes.

An enhanced RES-YOLO detection technique was presented for automated vehicle detection [29]. The proposed method enhances the detection performance of the conventional YOLO method by selecting optimum-feature networks and constructing adaptive-loss functions. The YOLO deep-learning vehicle-identification model was created and was then contrasted with current sophisticated target-recognition techniques.

This study seeks to create a path from the present to the future by establishing an efficient path. Compared to the current protocol, it produces the shortest route, thereby reducing packet loss, increasing throughput, and decreasing latency. By avoiding congestion, all current routing algorithms attempt to increase throughput and decrease end-to-end latency. The suggested method, on the other hand, increases throughput by decreasing end-to-end delay and increasing energy economy. Furthermore, the suggested algorithm not only manages congestion but also detects it automatically. Table 1 summarizes and compares the previously mentioned contemporary approaches.

## 3. Proposed Heterogeneous Algorithm for Efficient-Path Detection

In this section, we first present the general framework of the proposed heterogenous algorithm, called ACO-PT, and then we present each algorithm used, including ant-colony optimization and pheromone termite. Moreover, we elaborate the congestion-avoidance module and the automatic-vehicle-detection module, which are the main components of the proposed ACO-PT.

### 3.1. Overview of the Proposed System

**Figure 2:**
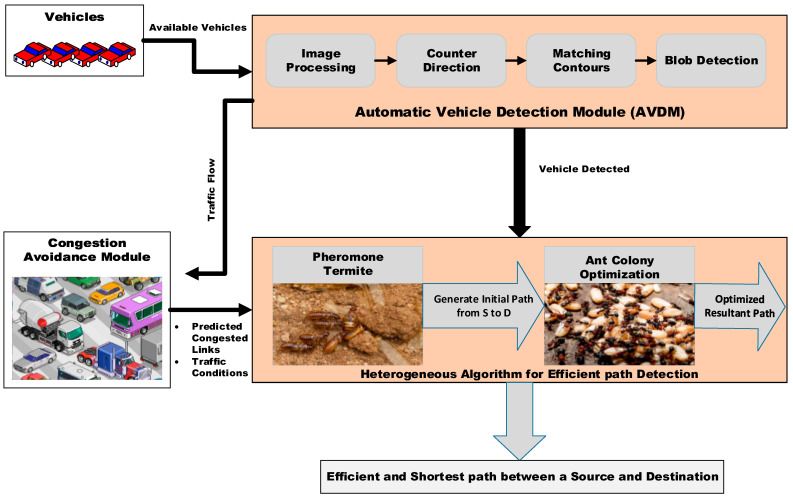
General framework of the proposed algorithm.

Our heterogeneous algorithm combines the concept of both ant-colony optimization and pheromone termite to find the most efficient and the shortest path between a source and a destination by using the pheromone information left by termites to guide ants’ pathfinding and develop solutions. Moreover, a congestion-avoidance module, automatic-vehicle-detection module, pheromone termite, and ant-colony optimization can work together as a unit to improve traffic flow and reduce congestion in a city. Figure 2 below illustrates the entire process and the general framework of our approach, including the modules and how these technologies can be integrated.

### 3.2. Deployment of Pheromone-Termite Algorithm for Vehicle Management

Routing in urban areas is highly critical because it increases latency and congestion. Consequently, the throughput performance of the network decreases. In this paper, a scalable mobility-aware pheromone-termite analytical model is used to provide robust and faster routing for improved throughput and minimum latency [30]. PT also provides support for network scalability and node mobility. In general, the pheromone-termite algorithm consists of using the process of real termites in search of food.

Pheromones are chemical compounds produced and released by organisms and are used for communication and coordination within a species. In the case of termites, pheromones are used to communicate information about food sources, potential mating partners, and colony defense. Termites use pheromones to coordinate the behavior of the colony and to maintain the social structure of the colony. For instance, when a termite finds a food source, it will lay down a pheromone trail that other termites can follow to the food source. This operation in termite colonies is a highly sophisticated and coordinated behavior that is essential for the survival and prosperity of the colony.

Termites use complex pheromone-communication processes. They produce pheromones in specialized glands and release them into the environment. Pheromones are dispersed through the colony and surrounding area through air or by being carried on termites. Other termites detect pheromones through receptors on their antennae, interpreting the pheromone trail as a signal to follow and take appropriate action.

In our case, the PT algorithm is used for initial distance estimation to generate the path between the source vehicle at the city entrance and the destination at the city exit. PT plays an important role because it assigns a value to each vehicle $V_{iv}$, which is denoted as:

(1) $$V_{iv}=E_{\left(e\right)}+T_{\left(V\right)}$$

where $E_{\left(e\right)}$ is the value of the trail between the source vehicle and the destination vehicle, and $T_{\left(V\right)}$ is the empirical estimation value of the most efficient path from the source vehicle.

The PT algorithm determines the most efficient path to reach the destination (vehicle). When the empirical estimation of the ideal path is successful, then the estimated value is properly confirmed.

**Figure 3:**
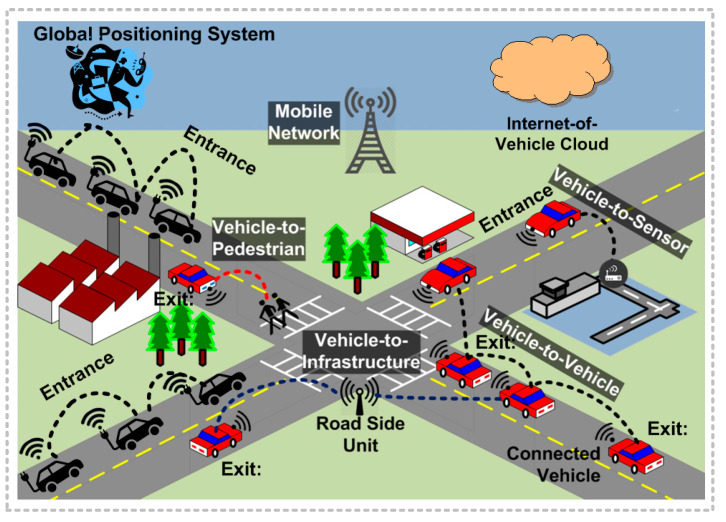
PT algorithm to handle entrance-and-exit processes.

The layout design of the vehicle scenario depicted in Figure 3 allows the PT algorithm to initialize $E_{\left(e\right)}$, which shows the estimated value of a given path from the entrance point to the exit point. A priority queue is performed to analyze the frequent choices for the vehicles, from the lowest value to the largest. The priority queue is considered the open set. Vehicles at all steps with lower values than $V_{iv}$ are unselected from the priority queue and then assigned values of *V* and *T*, and neighboring vehicles are chosen and computed accordingly. As mentioned, the neighboring vehicles are part of the priority queue. Thus, the PT algorithm continues searching until it reaches the vehicle that possesses the smallest value in the priority queue that is less than *V*.

### 3.3. Deployment of Ant-Colony-Optimization Algorithm for Vehicle Management

Ant-colony optimization is a heuristic optimization algorithm inspired by the behavior of ant colonies and is intended to approximate solutions for optimized problems. The algorithm simulates the behavior of ants as they search for food, where the ants leave pheromone trails that other ants can follow to find food. The algorithm uses this concept to find the best path through a problem space.

**Figure 4:**
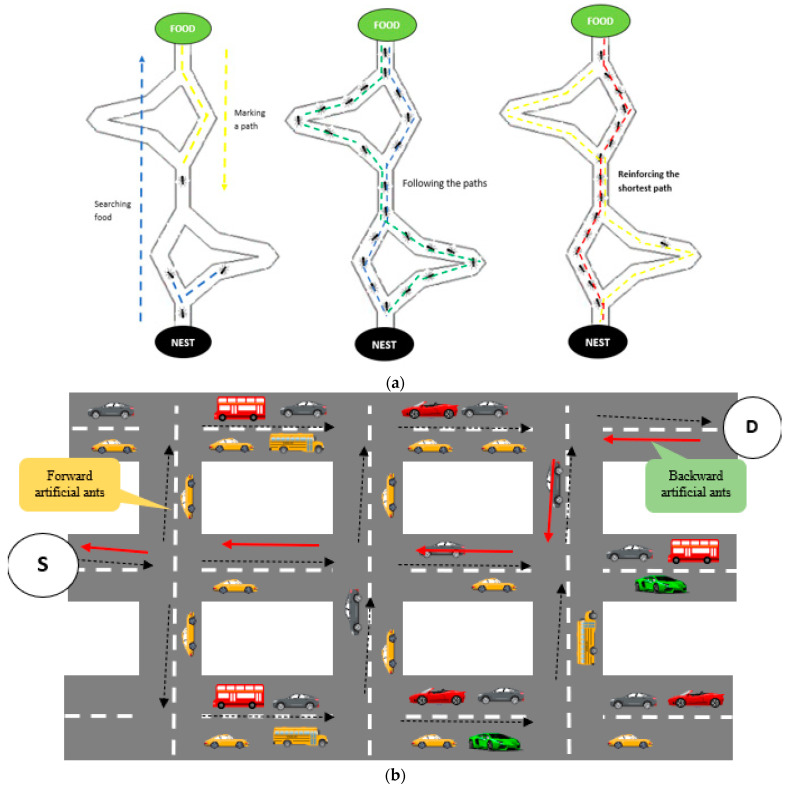
(**a**) Process of ant-colony optimization, (**b**) vehicle management following the ACO process to select the optimal route.

The general process of ant-colony optimization involves several steps. First, the algorithm starts by randomly placing a certain number of artificial ants on the problem space. Each ant is initialized with a random starting point and a set of rules for moving through the problem environment. Then, the ants select the next move based on a combination of heuristics and pheromone information. As they move, they construct solutions to the problem by building a path. They also deposit pheromones on the edges (routes). The number of pheromones deposited is determined by the quality of the solution the ant has found. After all the ants have found their solutions, the algorithm selects the best solution. Moreover, the pheromones on the edges evaporate over time, simulating the fact that pheromones tend to dissipate over time in nature. Finally, the algorithm continues to repeat these steps, with the ants updating their solutions based on changing pheromone levels, until a satisfactory solution is found or a maximum number of iterations is reached. Figure 4 illustrates the vehicle-management process.

In our case, the ACO algorithm optimizes the resultant path from the pheromone-termite algorithm to create the most efficient path that starts from the source vehicle and ends at the destination vehicle. The ant randomly pursues the food and returns to the colony. The ants spot its path using pheromones that can help other ants follow the same path. The chosen path has the highest number of pheromones that lead to an immediate search for food. Therefore, the value is identified based on the pheromones used by the ants.

In our proposed algorithm, food is assigned a value of (0, 1). If there is no vehicle entering the road, then the status is assigned a value of (0), which denotes food for ants. In the case of maximum (congested) vehicles on the road, then the status is (1), which denotes no food. In another perception, the ants apply the pheromones for path detection when they travel for food. Nevertheless, these pheromones disappear over time if a path is not in use. This situation demonstrates that the ants have started trailing alternate paths in search of food.

### 3.4. Heterogeneous ACO-PT Algorithm for Vehicle Management

The proposed heterogeneous ACO-PT algorithm is more efficient in pathfinding due to the combination of two cutting-edge algorithms. It is based on the behavior of real ants and on real termites in nature. At the beginning, the proposed algorithm starts, thanks to the features of PT, by selecting vehicles and assigning values to them. Then, it checks whether an ant has arrived at the target vehicle; if not, it restarts. Otherwise, it assigns an efficient path. Next, it assigns initial parameters, sets heuristic and distance matrices, locates the path for ACO, and determines the most efficient route for iteration.

The algorithm also takes into account the congestion-avoidance module and automatic-vehicle-detection module to find the most efficient path.

This progression assumes that ants use the most efficient path for food. Hence, ants are considered numbers and pheromones values in this model. The values are changed based on the usage of the path by ants. Subsequently, when ants discover the empty entrance on the road suggested by the PT algorithm, they initiate optimization according to specific procedures. ACO begins by initializing its provided specifications, which are the vehicle and ant counts. The pheromone creates the starting route.

**Figure 5:**
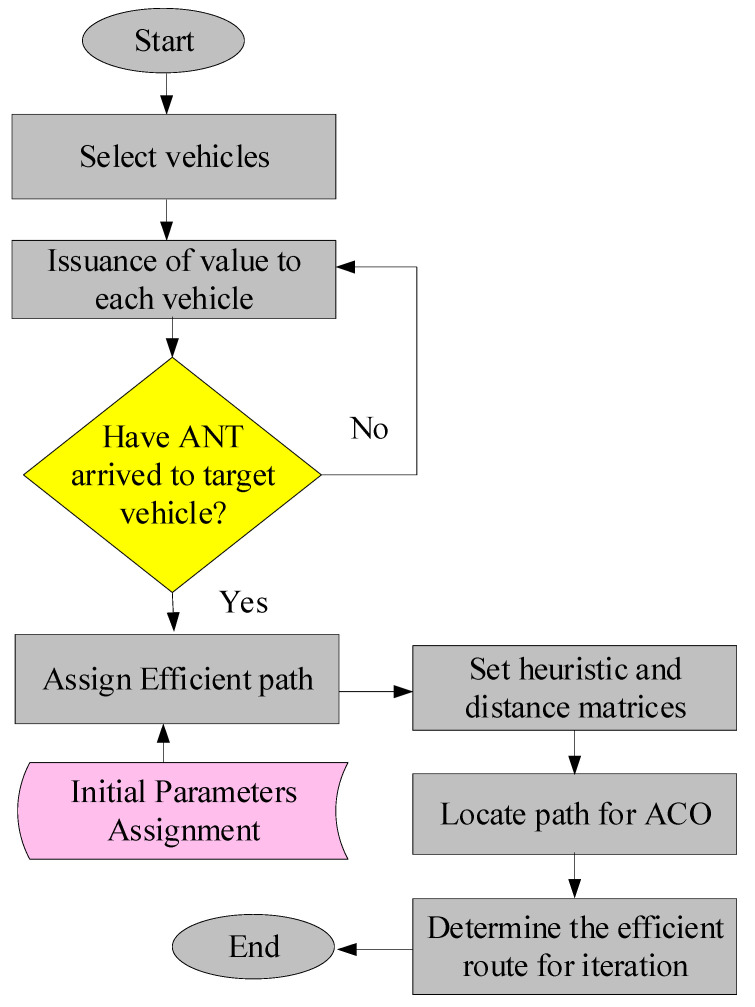
Heterogeneous algorithm for efficient-path detection.

The ACO forms the distances between matrices and vehicles. When all the parameters are complete, then an ant that is β is assigned a value of 1 and given to the initial vehicle that is at the entrance used by the vehicle. The ant begins from the first vehicle and provides random numbers from 0 to 1. The assigned random number is illustrated as 0.5. If this given random number is less than the initial path, then $\rho_{o}$. This illustration proves that there is a path available that will be followed by the ant. The heterogeneous algorithm for efficient-path detection is depicted in Figure 5. Algorithm 1 gives the pseudocode of the proposed heterogeneous ACO-PT algorithm, which consists of the following steps:
**Algorithm 1:** Deployment of ACO-PT for efficient-path detection**Initialization:** {V: vehicles; S: system;$\;E_{ri}$: efficient route for iteration;$\;V_{t}$: target vehicle;$\;P_{e}$: efficient path; An: ant; P: assign priority; $t_{c}$: time constraints; fu: fuel consumption; $V_{tp}$: vehicle type; $An_{a}$: artificial ant;$\;L_{s}$: location start; Ψ: initial parameters;$\;An_{t}$: total number of ants; γ: pheromone evaporation rate; ϓ: pheromone deposit rate; σ: heuristic matric; ω: distance matric; ϔϕ: pheromone interpretation; μ: most efficient path reaching the targeted vehicle using ACO}Input: { V, $V_{t}$}
**Output**: {$\;P_{e}$}
Set V $\mathrm{Identify}\;V\to S\in V_{t}$Assign$\;P\leftarrow \left(t_{c}+fu+V_{tp}\right)$
Place$\;An_{a}\forall ↔L_{s}$
$\mathrm{If}\;An\neq V_{t}$ then
  Set Ψ = $\left(An_{t}+\gamma +ϓ\right)$
  Restart Ψ 
$\;\mathrm{If}\;An≅V_{t}$ then    Set σ && ω       **Set** **ϔϕ**
    Locate μ→ACO$\;\;\;\;\mathrm{Assign}\;P_{e}$  **end if****end if**

Algorithm 1 uses ACO-PT to discover paths efficiently. The variable-initialization method for this algorithm is shown in Step 1. Steps 2 and 3 provide the input and output, respectively. Steps 4 and 5 begin the vehicle-selection process by identifying all vehicles in the system that must reach the target vehicle. Each vehicle is allocated a value and a priority depending on parameters such as time limits, fuel usage, and vehicle type. Step 7 depicts the arrival of the desired automobile. Each vehicle’s starting point is marked with the number of fake ants. The ants are utilized to create solutions to problems by constructing a trail across the surroundings. Steps 8 and 9 show that, if no ants arrive at the target vehicle, initial parameters for the ACO algorithm, such as the number of ants, the pheromone evaporation rate, and the pheromone deposit rate, are assigned. Step 10 depicts the restarting of the ACO-PT algorithm. Step 11 determines whether an ant has arrived at the target vehicle and provides the most efficient path. Step 12 explains the process of creating heuristic and distance matrices, as well as the heuristic and distance matrices that were used to aid the ants’ pathfinding. The pheromone-interpretation method is demonstrated in Step 13. As the ants proceed, they detect and analyze the termite-pheromone trails. This pheromone information may be utilized to direct ants to regions of the environment where termites have discovered food sources, indicating that these locations may be more efficient routes. Step 14 finds the most efficient path for all vehicles to reach the targeted vehicle using ACO, which is led by the pheromone information released by the termites. Step 15 determines the most efficient route for repetition, and the method is iterated several times to improve the path efficiency. When a good solution is found or the maximum number of iterations is achieved, the method is terminated.

Each ant can build a vehicle route using ACO, in which each vehicle visits each point. To select the next point ϐ, the ant can use a probabilistic formula.

Using ACO, each ant may construct a vehicle path in which each vehicle visits each location. The ant can apply probability formulas to choose the next location ϐ.

(2) $$ϐ=\arg\;\max\left\{\left(P_{A\left(\varepsilon ί\right)}\right){\left({Ď}_{\left(\varepsilon ί\right)}\right)}^{G}\right\}\;$$

where $P_{A}$ is the number of pheromones on the chosen path, ε is the current location, ί is the probable location, ${Ď}_{\left(\varepsilon ί\right)}$ is the inverse distance between two vehicle locations, and G establishes the relevance of distance in contrast to pheromone quality.

Locations previously visited by the vehicle are saved in working memory $M_{ŵ}$ and are not evaluated for selection. The value Ŕ represents a random uniform variable [0.1], whereas the value Ŕo represents a parameter. When each choice is made, the vehicle chooses the path with the highest rate selected from ϐ unless Ŕ is greater than Ŕo. In this case, the vehicle chooses a random variable Č, the next location to visit based on the probability distribution $P_{di}$, which favors short pathways with high pheromone levels.

(3) $$P_{di}=\frac{\left(P_{A\left(\varepsilon ί\right)}\right){\left({Ď}_{\left(\varepsilon ί\right)}\right)}^{G}}{\sum_{ί\neq Ŕ}\left(P_{A\left(\varepsilon ί\right)}\right){\left({Ď}_{\left(\varepsilon ί\right)}\right)}^{G}}\;if\;ϐ\neq Ŕ,\;\mathrm{otherwise}\;0\;$$

Using ϐ and $P_{di}$, each vehicle may take the most advantageous path that has already been created or choose a course at random based on a probability distribution determined by distance and pheromone buildup. If the vehicle-capacity limit is reached, the vehicle returns to the depot before proceeding to the next site. This technique is repeated until each area has been visited and the destination has been reached.

The pheromone trails of the vehicles must be updated to represent the vehicle performance and the caliber of the solutions discovered to enhance future solutions. This update is a crucial component of the adaptive-learning approach used by ACO and ensures that subsequent solutions will be better. After each individual solution has been created, a local update of the trail is performed, and after Ź solutions have been generated, a global update of the best route solution is performed.

To imitate the natural evaporation of pheromones and prevent any one path from becoming overly dominant, local updating is performed by lowering the quantity of pheromones on all paths that have been visited. The local trail update $Lt_{u}$ can be determined as follows:

(4) $$Lt_{u}=\left(1-e_{s}\right)P_{A\left(\varepsilon ϐ\right)}+\left(e_{s}\right)P_{A\left(0\right)}\;$$

where $e_{s}$ is the speed of evaporation and $P_{A\left(0\right)}$ is the initial pheromone value that is allocated to all paths.

Following the construction of a viable route by a set number of vehicles, a global trail update $G_{tr}$ is carried out by adding pheromones to each link in the efficient route discovered by a leading vehicle. The global trail update $G_{tr}$ can be calculated as follows:

(5) $$G_{tr}=\left(\left(1-e_{s}\right)P_{A\left(\varepsilon ϐ\right)}\right)G_{tr}+\left(e_{s}\right)*{\left(r_{o}\right)}^{-1}\;$$

where $r_{o}$ is the overall distance.

This update enhances the likelihood that future routes will use the pathways found in the best solutions and supports the usage of shorter best routes.

When necessary, PT finds new paths. When a vehicle lacks a valid routing-table entry and must send certain events or data to the main office, it creates a forward message and broadcasts it to all nearby vehicles. The local routing database of an intermediary vehicle that has received this forward message is searched for a viable route to the specified destination. If the search is successful, the receiving vehicle creates a backward data packet, which is then delivered through the reverse connections as a unicast message back to the source vehicle from which the initial request was made.

The vehicle establishes a reverse link to the node from which the forward message is received and broadcasts the forward data packet if it has no viable path to the destination. The destination node creates a backward data packet that is also broadcasted back to the source vehicle when it obtains the forward data packet. Each intermediary vehicle adjusts its routing table to build up a forward pointer upon receiving the backward data packet and then sends the backward message to its next hop by applying the reverse pointer. The procedure continues until the first source vehicle receives the reverse message.

When a packet reaches a vehicle, the pheromone for the source of the data packet is incremented by the reward $R_{e}$. The reward has a single nominal value. Only data packets sent to the vehicle are processed. If the vehicle is the recipient of the data packet at the next hop, this situation is addressed in advance. The pheromone-update process $P_{u}$ requires determining when a data packet is received at the source Sh vehicle from the previous hop Prh.

(6) $$P_{u}\left(Prh,Sh\right)=\left(P_{u}\left(Prh,Sh\right)\right)+R_{e}\;$$

The reward can be determined as follows:

(7) $$R_{e}=\frac{V_{tn}}{E_{in}-\left(E_{mi}-V_{n}\right)/\left(E_{av}-V_{n}\right)}\;$$

where $V_{tn}$ is the total number of vehicles, $E_{in}$ is the initial energy of the vehicles, $E_{mi}$ is the minimum energy of the path traversed by the forward lead vehicle, and $E_{av}$ is the average energy of the path traversed by the forward lead vehicle.

The nominal pheromone-evaporation interval is set to one second for the vehicle, which is known as the decay time, and it represents pheromone degradation, which is determined as follows:

(8) $$T_{ev}\left(ń,Д\right)=T_{ev}\left(ń,{Д}^{ef^{-v}}\right)\;$$

where $T_{ev}$ is entry into the PT for the vehicle, ń is the neighbor index for the vehicle, Д is the destination index for the vehicle, and $ef^{-v}$ is the evaporation factor.

Some vehicles need a slow rate of decay. To account for pheromone loss (1−ѽ), the percentage of the original value is regularly deducted from each value in the pheromone table, which can be illustrated as follows:

(9) $$T_{ev}\left(ń,Д\right)=\left(1-ѽ\right)T_{ev}\left(ń,Д\right)\;$$

where $k_{1}$ is the distance of the vehicle from the source in the horizonal axis and $k_{2}$ is the distance of the vehicle from the source in the vertical axis.

Each vehicle-routing table in PT is initialized with a uniform probability distribution $P_{r}$, which can be determined as follows:

(10) $$P_{r}\left(k_{1},k_{2}\right)=\frac{1}{V_{n}}\;$$

Upon arrival at vehicle s, an incoming packet with destination d is routed randomly based on the amount of d’s pheromone present on the neighbor links of s. A packet is never forwarded to the same neighbor from whom it was received—the previous node. If s has only one neighbor, i.e., the vehicle from which the packet was just received, the packet is dropped. Equation (6) details the transformation of pheromones for d on link s Ts,d into the probability Ps,d that the packet will be forwarded to d.

When a packet arrives with destination $k_{2}$ at a vehicle with source $k_{1}$, it is routed based on the number of pheromones present at source $k_{1}$ neighbor connections of the vehicle. A packet is never transmitted to the same neighbor, the prior vehicle, from which it was received. If the vehicle at $k_{1}$ has just one neighboring vehicle, namely, the vehicle from which the packet was received, then the packet is discarded. The pheromone for $k_{2}$ is on link $k_{1}$. Thus, the probability $P_{r}\left(k_{1},k_{2}\right)$ of transmitting the packets from the source to the destination can be determined as follows:

(11) $$P_{r}\left(k_{1},k_{2}\right)=\frac{{\left(T\left(k_{1},k_{2}\right)+ft_{p1}\right)}^{ft_{p2}}}{\sum_{i=1}^{V_{tn}}T\left(k_{1},k_{2}\right)+ft_{p1}{)}^{ft_{p2}}}\;$$

where $ft_{p1}$ and $ft_{p2}$ are the parameters that are employed to fine-tune the routing behavior of PT.

PT extends the life of a vehicle network $V_{lt}$, which demands the most remaining energy. The difference in total energy of the vehicle network and the sum of the average utilized energy of vehicles and their energy variation are used to forecast vehicle-network longevity.

(12) $$V_{lt}=VN_{te}-\frac{E_{c}}{V_{tn}}+E_{d}\;$$

where $VN_{te}$ is the total energy of the vehicle network, $E_{c}$ is the consumed energy, and $E_{d}$ is the energy deviation.

## 4. Congestion-Avoidance and Vehicle-Detection Modules of ACO-PT for Vehicles

Two innovative components make up ACO-PT that significantly aid in easing traffic and controlling the flow of vehicles.

▪ Congestion-avoidance module;
▪ Automatic-vehicle-detection module.

### 4.1. Congestion-Avoidance Module

In urban areas, road congestion has become a serious problem. Many researchers have started to use VANET techniques to detect potential congestion on roads. Nevertheless, these techniques have many limitations, such as channel-competition problems and delays [31].

Moreover, to enhance the efficiency of our approach in the search for the optimal path, a congestion-avoidance module was implemented. The purpose of this module is to effectively reduce vehicle fuel and energy consumption, decrease carbon emissions, and protect the environment. The combination of our ACO-PT algorithm with the avoidance-congestion module helps to avoid peak-hour traffic jams and temporal traffic blockages. The module consists of handling the congestion problem in the vehicle environment. To do so, we start by calculating the average speed of road movement, which depends on the density of vehicles. The latter reflects the number of vehicles per unit of road length. Additionally, the density of vehicles is inversely proportional to the speed of the movement of vehicles. The resultant linear relationship between density R and traffic velocity v is assumed as:

(13) $$R\left(v\right)=-av+R_{0}\;$$

where *a* is an arbitrary constant.

**Definition** **1.** *The movement of vehicles ordered by the transport network is called traffic flow. Indeed, the flow* F *is determined by the flow of vehicles per unit time as the product of* R:

(14) $$F\left(v\right)=-av^{2}+R_{0\;}$$

*where a is an arbitrary constant and* v *is the traffic velocity.*

**Corollary** **1.** *Traffic jams are formed in sections of the road where there is a high density of slow-moving vehicles with large traffic flow.*

**Proof.**  It is assumed that the flow of vehicles along street A ($Flow_{\left(A\right)}$) is greater than that along street B ($Flow_{\left(B\right)}$) in a certain time interval. □

**Hypothesis** **1.** *If the switching time is the same between all vehicles, then the occupancy of street A by transport depends on the outgoing* $Flow_{O\left(A\right)}$ *and incoming* $Flow_{I\left(A\right)}$ *traffic flow to the intersection of the streets within a green light.*

**Proof** **1.** Suppose that the waiting time for the vehicle is $t_{w}\;$ and the time for the green traffic light is $t_{g}$. Assume that M number of cars manage to cross the intersection in time $t_{g}$. At the same time, including waiting time for the green light $t_{w}$, $V_{n}$ is the number of vehicles arriving at this section of the street. Therefore, if for time $t_{w}+t_{g}$ the following expression is true: M<N, then the street is occupied by a high density of vehicles; otherwise, the traffic flow is considered free. □

**Figure 6:**
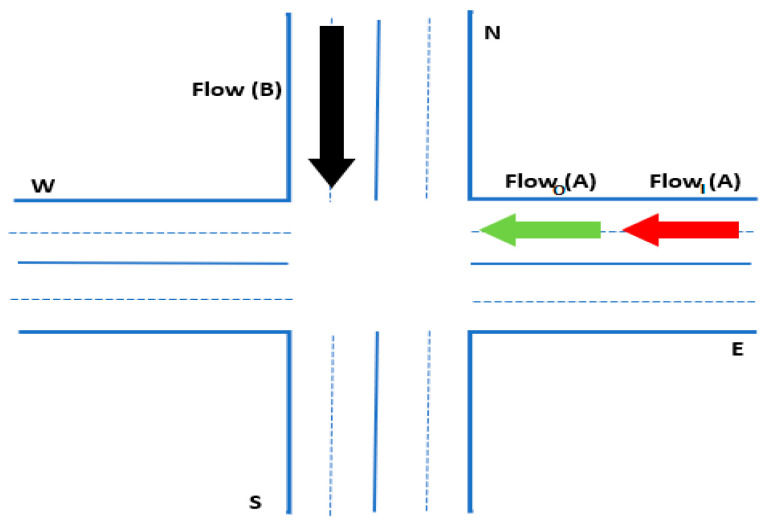
Incoming- and outgoing-traffic-flow illustration.

Figure 6 illustrates the situation at the intersection of streets A and B.

**Corollary** **2.** *At the intersection of two streets where traffic is controlled by the traffic system, the street with more traffic flow should receive more green-traffic-light time than the street with fewer vehicles. This expression is one of the conditions for avoiding traffic jams at street intersections.*

**Proof.**  $V_{at}$ be the average travel time for the vehicle on link $V_{pa}$. Thus, $T_{f}$ can be the free-flow time required on the given link $V_{pa}$ for the number of vehicles $V_{n}$. Therefore, congestion avoidance for the number of vehicles $V_{tn}\left(c\right)$ can be calculated as follows:

(15) $$V_{tn}\left(c\right)=T_{f}\left(1+\frac{\gamma }{ή}{\left(\frac{V_{n}}{C_{rv}}\right)}^{ή}\right)\;$$

where γ is the allocated route, ή is the set of routes, and $C_{rv}$ is the congestion rate for the vehicles. □

Therefore, the flow of traffic $V_{tn}$ should be less than the congestion rate for the vehicles $C_{rv}$. Therefore, $\frac{V_{tn}}{C_{rv}}$ can be small. Thus, the average travel time for the vehicle is equal to the free-flow time, which can be expressed as $V_{tn}\left(c\right)\approx T_{f}$. For the number of vehicles $V_{n}$, the impacts of congestion begin to be observed.

### 4.2. Automatic-Vehicle-Detection Module

**Figure 7:**
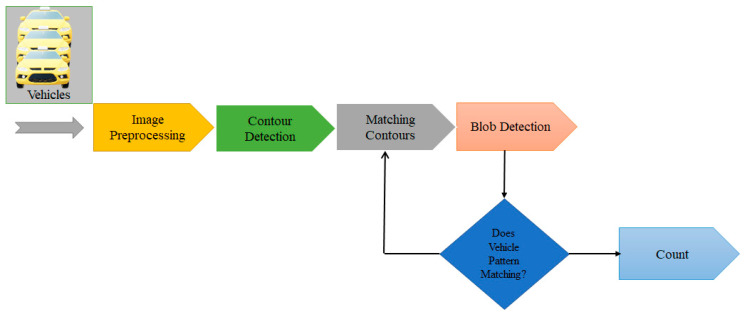
Automatic-vehicle-detection process.

The automatic-vehicle-detection module is used to detect the presence and movement of vehicles in a specific area. The AVD module typically consists of several components, including sensors, cameras, and software that work together to detect and track vehicles. To meet the efficient-path condition, it is necessary to automatically detect the traffic. For this purpose, the vehicle-detection module consists of four stages, depicted in Figure 7, and the automatic vehicle detection is explained in Algorithm 2:

- Image preprocessing;
- Contour detection;
- Matching contours;
- Blob detection.

**Algorithm 2:** Automatic-vehicle-detection process**Initialization (A):** {$src_{i}$: matrix (source); $dst_{i}$: binary image (destination), $I_{st}$: street-image frames; Ť: threshold transformation; Ἇ: edge detection (amplitude), ∃ϵ: steepest; $I_{gr}$: image gradient;$\;E_{h}$: horizonal edges, $E_{s}$: edges Sobel; Ĝ: Gaussian filter; $I_{n}$: image noise; ∻: reduction; $O_{c}$ contours objects; Ḻ: Laplace operator; $S_{ir}$: shapes irregular; Ar: area; $R_{as}$: aspect ratio}$\mathrm{Input}:\;\{\;I_{st}$}**Output: {**Ἇ**}**//*Image preprocessing$\mathrm{Receive}\;I_{st}\leftarrow src_{i}$**Apply** Ť on $dst_{i}$
$\mathrm{Determine}\;I_{gr}\Leftrightarrow \exists \epsilon$ 
$\mathrm{Detect}\;E_{h}\;\&\&\;E_{s}\;$**Confirm** Ἇ//*Contour detectionApply Ĝ→($∻(I_{n}$))
$\mathrm{Detect}\;O_{c}\;┴\;Ḻ$ 
$\mathrm{Filter}\;O_{c}\leftarrow \left(Ar+R_{as}\right)\propto S_{ir}$//*Marching contours$If\;O_{c}=PO_{c}$ thenFind V
else$\mathrm{discard}\;O_{c}\;\&\&\;\;V\neq V_{t}$end else**end if**//*Blob detectionInitialization (B): ℵ: brightness; $A_{sr}$: surrounding area; $C_{b}$: brighten color; $T_{g}$: geometric transformations; $\ddot{\hat{T:}}$ track;$\;\overset{ˇ}{D}:$ discard;$\;V_{P}$: vehicle position; Wd: width; Ht: height}
$\mathrm{Apply}\;Ĝ\left(I,\mathrm{II}\right)\approx \left[C_{b}+ℵ+A_{sr}\right]$$\mathrm{Apply}\;T_{g}\neg \left(\ddot{\hat{T,}}\overset{ˇ}{D}\right)\to \left(V_{P}+Wd+Ht\right)$Detect V↔I

Algorithm 2 demonstrates the vehicle-automation process. Step 1 initializes the variable for preprocessing, contour detection, and matching contours. Steps 2 and 3 show the input and output, respectively. Step 4 demonstrates the receiving process of the street-image frame given by matrix ($src_{i}$). Step 5 applies a threshold transformation to create a binary image ($dst_{i}$). Step 6 determines the direction of the steepest increase by the image gradient. Step 7 shows the detection process of the horizontal edges and the Sobel edge direction. Step 8 summarizes the edges to determine the approximate amplitude of the Sobel detector. Step 9 shows the image-noise reduction process by applying the Gaussian filter. Step 10 detects the contours of objects by applying the Laplace operator. Step 11 filters the contours, which are based on their area and aspect ratio, to remove small or irregular shapes. Steps 12 and 13 show the contour-matching process and compare the filtered contours to a set of predefined contour templates to determine if they match a vehicle, respectively.

Steps 14 and 16 show the discard process for contours that do not match a vehicle. Step 17 initializes the variables for blob detection. Step 18 applies the first and second derivations of the Gaussian filter to brighten the color and determine the brightness and surrounding areas. Step 19 applies geometric transformations to discard and track vehicle position, width, and height. Step 20 shows the outcomes of the location for each detected vehicle in the image.

#### 4.2.1. Image Preprocessing

The first stage consists of preparing the image for further processing. It typically includes a series of operations, such as image enhancement and noise extraction. These operations are applied to improve image quality and extract useful information. The goal of image preprocessing is to make the image more suitable and useful for the recognition and detection of vehicles. First, the camera receives the street-image frame provided by the matrix $src_{i}$. To remove uninteresting pixels from the image, a binary-threshold transformation is applied so that only pixels that are above or below a certain value remain in the final image ($dst_{i}$).

(16) $$dst\left(x,y\right)=\{\begin{array}{c} 1,\;\;if\{\begin{array}{c} \left|I_{t}\left(x,y\right)-I_{i+1}\left(x,\;y\right)\right|>T\\ M \end{array}\\ 0,\;elsewhere \end{array}\;$$

where $I_{t}$ is an initial street image with the *i*-th pixel; dst(x, y) is an image after thresholding; T is the threshold value, chosen for sufficient detection of contours in the original image; and M is the maximum pixel value of the threshold transforms.

**Definition** **2.** *The gradient of the image determines the direction of the steepest increase in a certain value.*

(17) $$\left|grad\;g\left(x,\;y\right)\right|=\sqrt{{\left(\frac{\partial f}{\partial x}\right)}^{2}+{\left(\frac{\partial f}{\partial y}\right)}^{2}}\;$$

**Property** **1.** *Edges are normal to the direction of the gradient and can be classified by a light profile in the direction of the gradient at a given pixel* ɷ*, which can be determined as:*

(18) $$ɷ=arg\left(\frac{\partial f}{\partial x},\;\frac{\partial f}{\partial y}\right)\;$$

**Definition** **3.** *To detect horizontal edges, calculate the difference between a value of one pixel and the next pixel value, up or down from the first, starting from the top-left origin:*

$$H_{c}=diff_{y}\left(x,y\right)=value\left(x,y\right)-value\left(x,y+1\right)$$

*Likewise, equivalent row detections can be illustrated as follows:*

(19) $$H_{R}=diff_{x}\left(x,\;y\right)=value\left(x,y\right)-value\left(x-1,\;y\right)\;$$

*where* $H_{c},\;H_{R}$ *are column and row detectors, respectively.*

**Definition** **4.** *Sobel edge detection selects all edges of an image regardless of direction.*

(20) $$M=\sqrt{\left(h_{1}^{2}+h_{2}^{2}\right)}\;$$

**Property** **2.** *The approximate amplitude of the Sobel detector is a summation of the edge module:*

(21) $$M=\left|h_{1}\right|+\left|h_{2}\right|\;$$

*where* M *is a Sobel edge detector and* $h_{1}$ *and* $h_{2}$ *are the edges.*

#### 4.2.2. Contour Detection

After enhancing the image quality, contour detection is used to detect and extract the shapes of objects in an image. It aims to find the boundaries of objects within an image. Contours are defined as the boundaries of an object, represented by a set of points in an image. These points can then be used to create a representation of the vehicle. Since all contour-detection results are easily affected by noise in the image, it is vital to filter out noise in the image to decrease false detection. The Gaussian filter is applied for the smooth image ${Š}_{im}$, which can be given by:

(22) $${Š}_{im}=\frac{1}{2\pi \sigma }\exp\left(-\frac{{\left(i-\left(k_{1}+1\right)\right)}^{2}+{\left(j-\left(k_{2}+1\right)\right)}^{2}}{2\sigma^{2}}\right)$$

where σ is the standard deviation, $k_{1}$ is the distance of the vehicle from the source on the horizonal axis, and $k_{2}$ is the distance of the vehicle from the source on the vertical axis.

The second stage uses the Laplace operator to detect the contours of objects Laplace(f), which can be determined as follows:

(23) $$Laplace\left(f\right)=\frac{\partial^{2}f}{\partial x^{2}}+\frac{\partial^{2}f}{\partial y^{2}}$$

where $\frac{\partial^{2}f}{\partial x^{2}}$ is the second derivative of the image pixels.

An edge in an image may point in a variety of directions, so the improvement in the Laplacian-operator Canny method with image filters to detect vertical, horizontal, and diagonal edges in the blurred image $B_{im}$ can be determined as follows:

(24) $$B_{im}=\sqrt{G_{x}^{2}+G_{y}^{2}}\;$$

From this calculation, the edge gradient and direction can be determined:

(25) $$\theta =\tan^{-1}(G_{x}^{2}+G_{y}^{2})\;$$

where G*_x_* is the first derivative in the horizontal direction, G*_y_* is the vertical direction, and ϴ is the edge gradient.

#### 4.2.3. Matching Contours

The goal of matching contours is to determine whether a contour in the image corresponds to a predefined contour template of vehicles. First, matching contours entails geometric matching that compares the shape and size of contours. Then, this method performs structural matching to compare the relations between contours, thus matching based on appearance to compare the texture, color, or other features of the contours. The result of the matching process is a similarity score, which indicates how similar the contour in the image is to the predefined template. The score is usually represented as a value between 0 and 1, where a value close to 1 indicates high similarity and a value close to 0 indicates low similarity. To do so, each contour found is checked for similarity to the image of the vehicle. For this purpose, a database of vehicle contours is prepared and predefined in advance. The easiest way to compare two contours is to calculate the contour moments. Roughly speaking, the moment is a rough characteristic of the contour, calculated by integrating (or summing) all the pixels of the contour. In general, the moment of contour (*p*, *q*) is defined as follows:

(26) $$m_{p+q}=\overset{n}{\underset{i=1}{\sum }}i\left(x,y\right)x^{p}y^{q}\;$$

where *p* is the order of *x* and *q* is the order of *y*.

The term order is employed, which indicates the degree to which the component of the sum is raised. Summation is carried out over all pixels of the contour boundary.

The integrity of the contours is critical for obtaining an accurate automated-detection method.

The selection of contours indicates that each contour is limited to being an integral unit in matching. Let each contour integrity be $C_{V}^{I}$ for the vehicle that requires edge pixels $P_{i}^{\left(v\right)}$. Hence, there are two options: Either all of the edge pixels participate in the matching or none of them does. Contours that partially match are not permitted. The same constraint is employed to model contours $C_{v}^{M}$. The contour-selection indicators are introduced, which should be written as $A_{I}^{sel}\in {\left\{0,1\right\}}^{\left|1\right|\times 1}$ in the whole vehicle image and $B_{I}^{sel}\in {\left\{0,1\right\}}^{\left|M\right|\times 1}$ in the vehicle modeling. Thus, an image for vehicle-contour-integrity selection and a model for vehicle-contour-integrity selection can be determined as follows:

(27) $$A_{I}^{sel}=\{\begin{array}{c} 1\;\mathrm{contour}\;C_{V}^{I}\;is\;selected\\ 0\;otherwise \end{array}\;$$

(28) $$B_{I}^{sel}=\{\begin{array}{c} 1\;\mathrm{contour}\;C_{V}^{I}\;is\;selected\\ 0\;otherwise \end{array}\;$$

#### 4.2.4. Blob Detection

To improve image recognition, blob detection aims to detect and locate blobs (i.e., regions of interest) in an image. Blobs are typically defined as regions of an image that are distinguishable from the surrounding pixels based on their color, texture, or intensity. We start by applying the first derivative of the Gaussian filter $G_{ft}$, which is inversely proportional to the size of dark pixels $D_{p}$ and can be calculated as follows:

(29) $$G_{ft}=\frac{1}{D_{p}\sqrt{2*\pi }}\;$$

The second derivative of the Gaussian filter, called the Laplacian operator, must be multiplied by *σ* to obtain the response-scale invariance of vehicle $\nabla_{norm}^{2}g$, which can be determined as follows:

(30) $$\nabla_{norm}^{2}g=\sigma^{2}(\frac{\partial^{2}g}{\partial^{2}x}+\frac{\partial^{2}g}{\partial^{2}y})$$

To obtain the best response, the Laplacian zeros denote that the pixel boundaries $B_{p}$ must be circle aligned, which is given by:

(31) $$B_{p}=(x^{2}+y^{2}-2\sigma^{2})e^{-\frac{x^{2}+y^{2}}{2\sigma^{2}}}\;$$

In this case, the maximum response or external area of interest pixels Ϛ in a scale can be determined as follows:

(32) $$Ϛ=\frac{r}{\sqrt{2}}\;$$

where r is the distance between pixels.

Due to some additional factors, such as occlusions and shadows, the constructed blob model might give inappropriate results. To obtain this model, additional information must be supplied: position, width, and height. Additionally, geometric transformations should be applied to discard vehicle positions in inappropriate places. This computation obtains the distance of the vehicle blob with pixel coordinates (x, y) for an appropriate distance $r_{ap}$ and can be obtained as follows:

(33) $$r_{ap}=h*\frac{f*\cos\theta +y_{v}*\sin\theta }{f*\sin\theta -y_{v}*\cos\theta }\;$$

**Corollary** **3.** *The duration of the intermediate cycle should be such that a car approaching a highway intersection on a green signal at the speed of free movement can either stop at the stop lines or have time to freely pass through the intersection when the signal changes from green to yellow. Considering the constant deceleration experienced when braking in front of the stop line, the duration of the intermediate cycle is given by:*

(34) $$t_{i}=\frac{v}{2*a}+\frac{l_{s}+l_{v}}{v}\;$$

*where* $t_{i}:$ *the intermediate cycle time,* v*: vehicle velocity, a: vehicle stopping acceleration,* $l_{s}:\;$*the length of the stop line, and* $l_{v}:$ *vehicle length.*

**Theorem** **1.** *In the period of the intermediate cycle, pedestrians who had previously crossed the carriageway at a permitting traffic light also end their movement. During time* $t_{p}$*, the pedestrian must either return to the sidewalk from where they began to move or reach the middle of the carriageway. The time for a pedestrian to cross the road is:*

(35) $$t_{p}=\frac{B}{4*v_{p}}\;$$

*where B is the width of the road and* $v_{p}$ *is the precalculated average speed of the pedestrian.*

This approach is based on image-processing methods that calculate the number of vehicles $V_{n}$ in both streets and set the appropriate traffic-light switching times accordingly.

**Definition** **5.** *Phase coefficients are necessary to determine the duration of the main traffic-light cycles of regulation:*

(36) $$y_{i}=\frac{N_{i}}{M_{j}}\;$$

*where* $y_{i}$ *is the phase coefficient of the given direction,* $N_{i}$ *is the traffic intensity for the considered period of time, and* $M_{j}$ *is the density of traffic flow in the given direction of the control phase.*

The duration of the regulation cycle should be calculated, which helps to determine the hop distance of the vehicle.

(37) $$T=\frac{\sum C_{i}}{1-\left(y_{1}+y_{2}+\ldots +y_{n}\right)}\;$$

where *T* is the duration of the intermediate cycle, $\sum C_{i}$ is the summation of all intermediate cycles, and $y_{n}$ is the phase coefficient.

Finally, the automated-vehicle-detection module is critical to the whole process because it provides precise and trustworthy information about each vehicle in the image, which is then utilized to determine the most efficient and the best path for the vehicles.

## 5. Experimental Results and Discussion

The proposed ACO-PT algorithm was simulated using the 3D VANET ns-3.37 simulator. The simulation was conducted on various congested scenarios. The performance of the algorithm was monitored while vehicles were transferred from one unit to another. To effectively manage the mobility of vehicles, the use of wireless-sensor networks that are mobility-aware was necessary. Realistic congested scenarios were employed to accurately assess the impact of the proposed ACO-PT algorithm.

VANET is a network without any infrastructure if we consider the network that is produced by automobiles. There is no need for a physical channel for vehicle-to-vehicle communication. No centralized regulatory body is necessary. VANET does not require switches or hubs since hop-to-hop communication is possible. Even though they are situated along the sides of the road, RSUs and TAs are only basic resources.

The performance of the proposed ACO-PT algorithm was compared to existing protocols such as ACO-FL, RACO, and AODV-ACO. It was observed that the performance of the proposed ACO-PT algorithm, as well as the competing algorithms, may be affected by the level of mobility and the number of hops involved in the network. WSNs experience several challenges and limitations related to mobility and scalability. To tackle these issues, the energy-efficient border-node medium-access-control (BN-MAC) protocol was employed [32]. The main goal of using BN-MAC was to reduce energy consumption while maintaining high levels of mobility, scalability, and collision avoidance at the second layer.

Wireless-sensor networks rely on bidirectional end-to-end dependability to ensure smooth data flow and efficient use of bandwidth. The lack of bidirectional dependability can affect the accuracy of event detection and data collection. To achieve end-to-end dependability, a bidirectional consistent transport system that uses a brief preamble acknowledgment/non-acknowledgment (ACK/NACK) control packet between vehicles was employed. To conserve energy, vehicles communicated with each other using short-range, one-hop communication instead of long-range communication. Multihop communication does not offer significant advantages over single-hop communication and can lead to increased delay as each node stores and sends packets. The simulation scenario included a maximum of 400 vehicles with a transmission range of 45 m placed randomly but in a consistent pattern within a 900 × 900 square-meter space.

Several testing scenarios were constructed, starting with 50 vehicles. There was no congestion with the utilization of 50 vehicles. Consequently, the number of vehicles was increased in each testing scenario, and congestion was detected with 300 vehicles. Thus, the suggested network could support a maximum of 400 vehicles, demonstrating a realistic situation. The dynamic link–origin–destination-matrix estimate (DLODME) characterizes vehicle traffic in a specific area to analyze transportation demand. DLODME supports modern technologies such as Bluetooth and the Global Positioning System (GPS) and allows for trajectory retrieval. Furthermore, it lowers the number of variables linearly rather than quadratically depending on network size. It also employs vehicle counts perceived by traffic-count sensors; it does not require past origin–destination trip data or any anticipated flow distribution for an estimate.

Simulation of Urban Mobility (SUMO) was employed to generate a random Manhattan-grid road network with random traffic flows and traffic signals. The SUMO traffic-simulation power was effectively integrated with NS3 network-simulation capabilities. The Online Vehicular Network Integrated Simulation (OVNIS) [33] platform was used to connect SUMO with NS3 such that the SUMO scenario was included in the NS3 mobility model. The NS3 module affected traffic simulation by rerouting vehicles in the mobility model. OVANIS addressed this issue by creating a submodule in NS3 utilizing the TraCI interface. SUMO created the vehicle-movement patterns needed by NS3 to evaluate the effectiveness of the proposed ACO-PT routing protocol.

The sensor node linked to each vehicle had an initial energy of 5 joules. The simulation aimed to reduce energy consumption and improve data delivery for vehicles by simulating different realistic conditions, such as movement and static conditions. The test findings were consistent with real-world experimental outcomes and revealed that more energy was required when forwarding packets through multiple hops; this situation was exacerbated when the packet size was larger. Each sensor node was configured with a bandwidth of 100 kbps/s and an electricity consumption of 18 milliwatts. The sensor nodes connected to the vehicles were able to broadcast data at a power level ranging from –18 to 15 dBm. The simulation was run for a total of 10 min, with 20 s pauses, and a total of 12 simulation runs were included in the analysis. The simulation parameters are summarized in Table 2.

To conduct the experiment, the following state-of-the-art dynamic vehicle scenario was generated:

Testing scenario: The dynamic vehicle scenario was employed, which demonstrated the traffic-participant behavior that was dynamically included in the simulation. The dynamic vehicle simulation simultaneously showed how a cooperative and autonomous vehicle behaves, including how its sensors are set up and how it drives. The conduct of the surrounding traffic participants was based on the status of the autonomous vehicle in the traffic simulation. A dynamic traffic environment was created that could respond to the driving behavior of the vehicle. Dynamic coupling guaranteed that cooperative and autonomous vehicles could operate in traffic. Every participant in a traffic flow had a changeable driver model.

To prevent significant step changes in the cooperative and autonomous sensor perception of the vehicle, this prediction was needed. According to the simulation results, the pause time gap in the simulation environment was sufficiently short for the results of the increased motion prediction to not significantly affect sensor perception. Additionally, this tendency is common for environmental modeling. It is customary to apply motion models during the actual sensor-fusion process, which creates a new perspective of the surrounding world. In the dynamic vehicle simulation, the simulated environment of the autonomous vehicle and its sensor models interacted. This paper does not delve into detail on how to improve cooperative simulations. Importantly, the infrastructure may be expanded in terms of collaboration simulations. However, the simulation environment of NS-3 can be increased so that it can simulate many cooperative and autonomous vehicles at once. The ability to assess aspects such as cooperative merging, traffic distribution, overtaking, etc., can be made possible by this capacity. The digital features of the twin are programmed and incorporated into the simulation environment to make it complete. It is crucial that the vehicle model accurately depict the realistic environment of the vehicles. Thus, it is possible to incorporate driving functions, including localization, trajectory planning, vehicle control, maneuver-intention prediction, and driving strategy. Based on the testing results, the following parameters were considered:

- Throughput;
- End-to-end delay;
- Energy consumption.

### 5.1. Throughput

**Figure 8:**
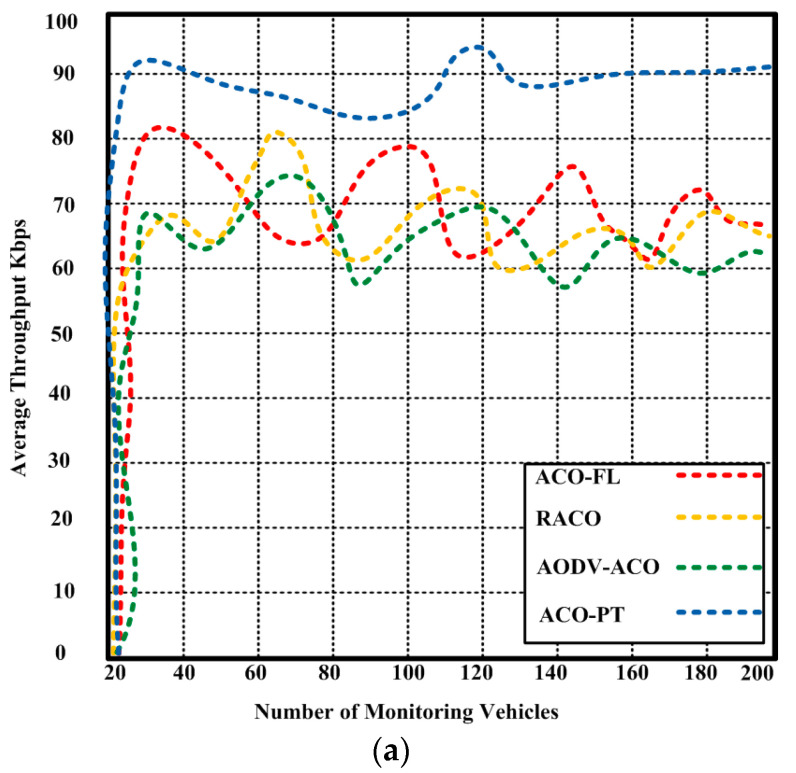

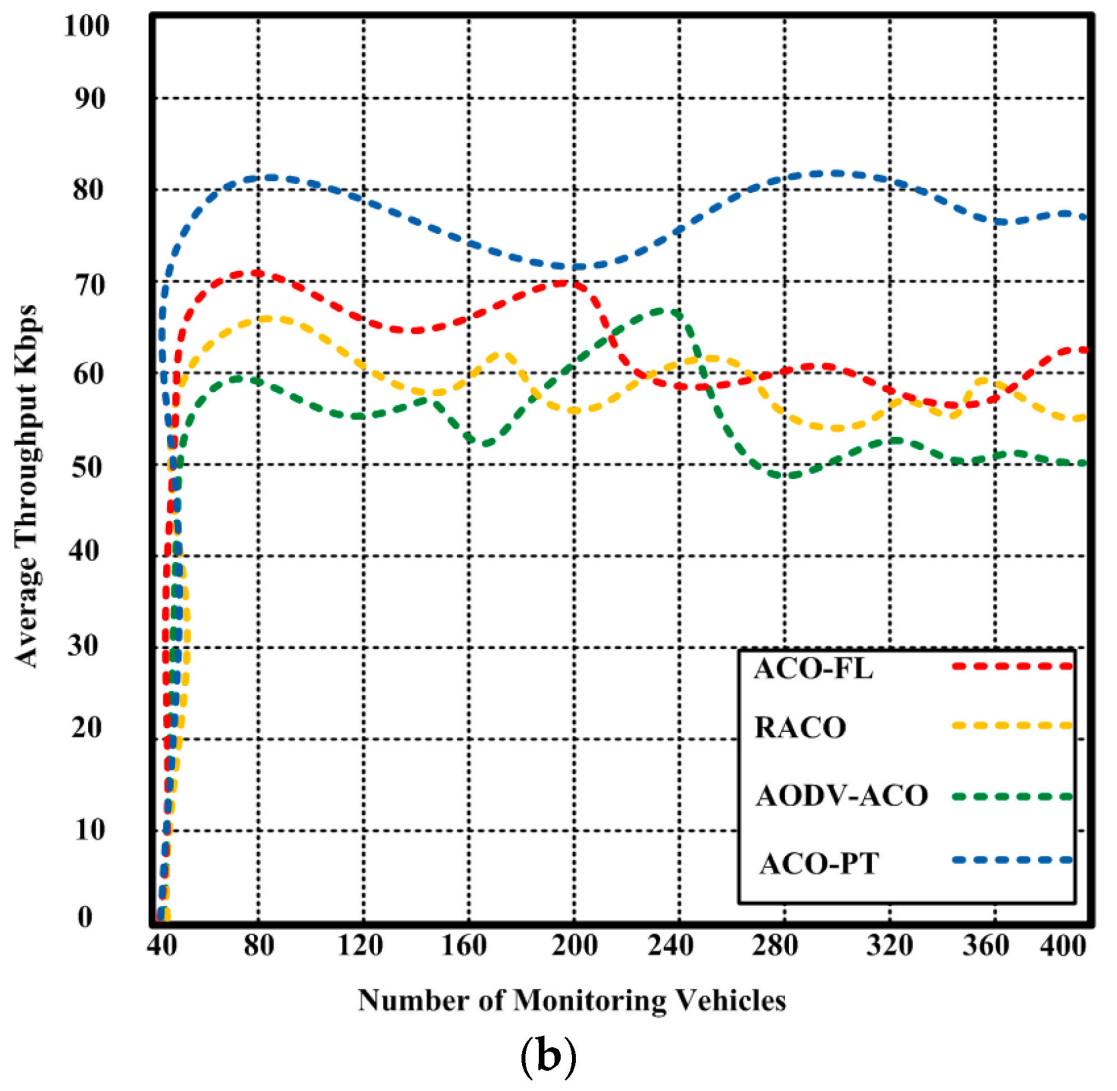
(**a**) Throughput of the proposed ACO-PT and contending algorithms (ACO-FL, RACO, and AODV-ACO) with a maximum of 200 vehicles, (**b**) throughput of the proposed ACO-PT and contending algorithms (ACO-FL, RACO, and AODV-ACO) with a maximum of 400 vehicles.

Throughput is a measure of the efficiency of the communication system in transmitting data; it is commonly used in networking and communication systems and can be measured in different layers and different ways. For this purpose and to validate the proposed ACO-PT, throughput was measured and compared to state-of-the-art algorithms ACO-FL, RACO, and AODV-ACO. Figure 8a shows that the proposed ACO-PT achieved a greater average throughput of 90.2 kbps with 200 cars than the competing algorithms, which achieved an average throughput of 62.1–68.5 kbps. When the number of cars was increased to 400 vehicles, the suggested ACO-PT, which obtained 78.2 Kbps, was not greatly affected. Figure 8b depicts the competing algorithms, which yielded 50–62.1 kbps. The usage of a congestion-avoidance module and an autonomous detection module resulted in greater throughput. An average throughput can be obtained as follows:

(38) $$T_{P}=\frac{\left(P_{s}*P_{si}\right)}{P_{dt}}\;T_{Pa}=\frac{V_{t}*T_{P}}{P_{dtt}}\;$$

where $T_{P}$ is the throughput of the vehicles, $P_{s}$ is the number of successful packets, $P_{si}$ is the size of the delivered packet, $P_{dt}$ is the total time for delivery of the packets, $V_{t}$ is the total number of vehicles, $P_{dtt}$ is the total time for the delivery of packets for all vehicles, and $T_{Pa}$ is the average throughput.

### 5.2. End-to-End Delay

**Figure 9:**
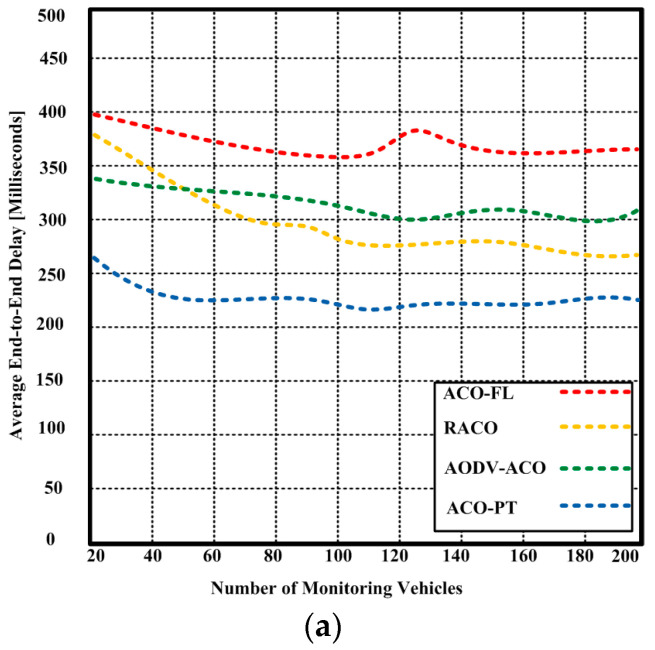

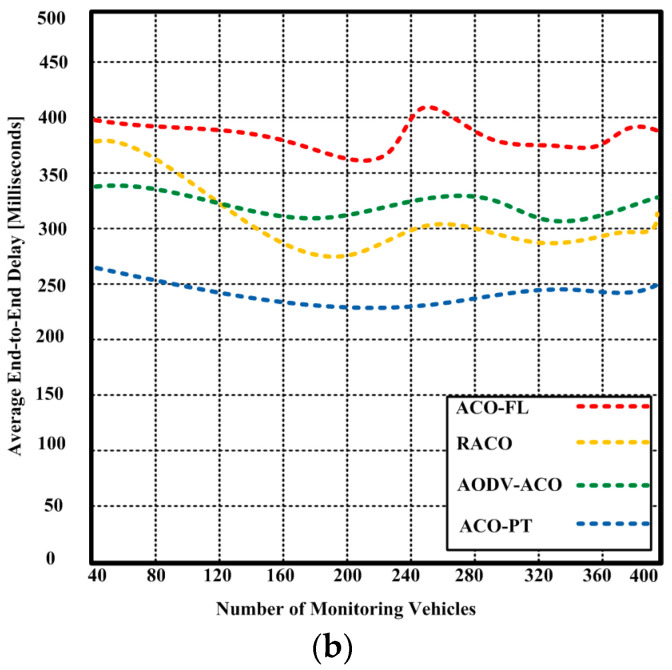
(**a**) End-to-end delay of the proposed ACO-PT and contending algorithms (ACO-FL, RACO, and AODV-ACO) with a maximum of 200 vehicles, (**b**) end-to-end delay of the proposed ACO-PT and contending algorithms (ACO-FL, RACO, and AODV-ACO) with a maximum of 400 vehicles.

To measure the quality of service (QoS) of the network, it is important to know the end-to-end delay or latency metric. This metric is crucial because it indicates how long it takes a packet to travel across a network from source to destination. Figure 9a displays the proposed ACO-PT and competing algorithms’ end-to-end latency (ACO-FL, RACO, and AODV-ACO). According to the testing data, the suggested ACO-PT had an end-to-end latency of 226.3 milliseconds with 200 vehicles. The competing methods, on the other hand, caused a substantially larger end-to-end latency of 262.3–363.3 milliseconds. When the number of vehicles was extended to 400, the end-to-end time for our suggested ACO-PT was similarly shorter than for other competing approaches, as depicted in Figure 9b. The suggested ACO-PT had a time delay of 250 milliseconds, whereas competing methods had a time delay of 300.8–388.7 milliseconds. It is concluded that the proposed ACO-PT algorithm was robust, resulting in lower end-to-end delay.

(39) $$E\_e_{d}=\overset{R}{\underset{i=1}{\sum }}\left(\frac{V_{d1}}{P_{r1}}+\frac{P_{si}}{T_{r}}\right)+\left(\frac{V_{d2}}{P_{r2}}+\frac{P_{si}}{T_{r}}\right)+\left(\frac{V_{d\left(V_{n}\right)}}{P_{r\left(V_{n}\right)}}+\frac{P_{si}}{T_{r}}\right)$$

where *R* is the total number of routers, $V_{d1}$ is the distance between vehicle 1 and vehicle 2,$\;V_{d2}$ is the distance between vehicle 2 and vehicle 3, $V_{dn}$ is the distance between $V_{n}$ vehicles, $P_{r1}$ is propagation rate 1, $P_{r2}$ is propagation rate 2, $P_{r2}$ is the propagation rate for *n* packets, and $T_{r}$ is the transmission rate.

### 5.3. Energy Consumption

**Figure 10:**
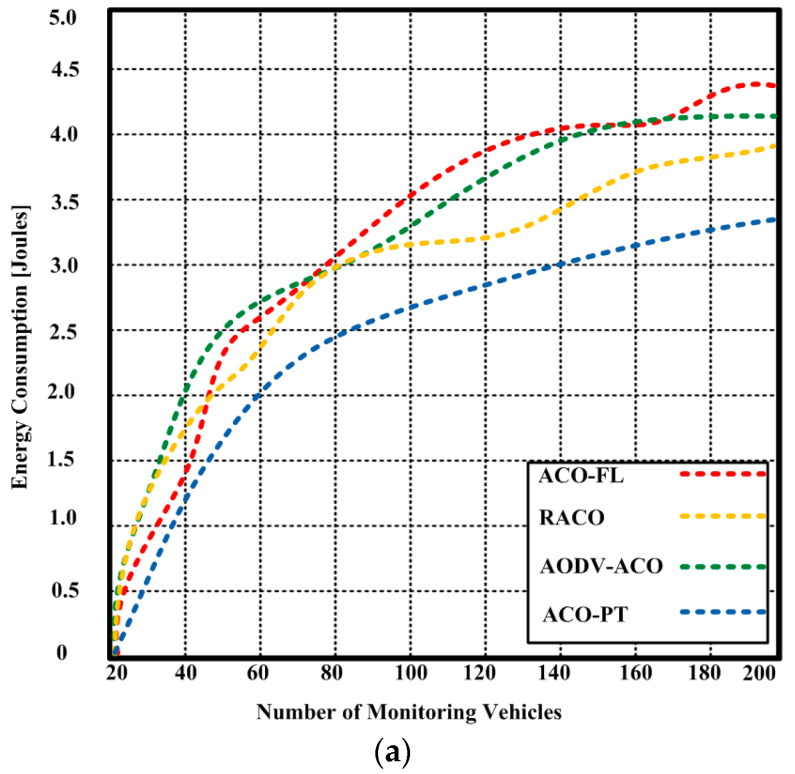

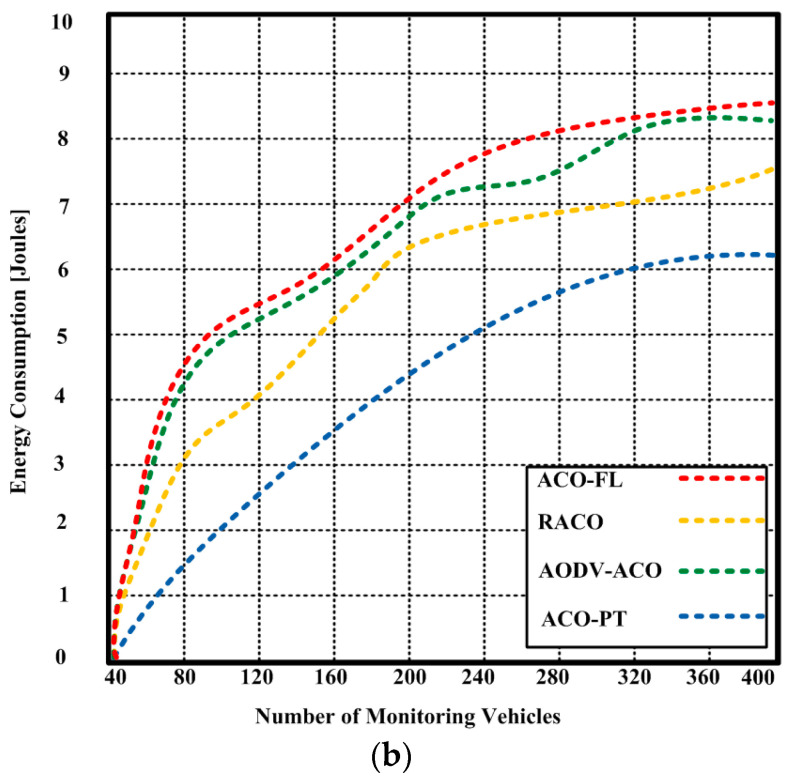
(**a**) Energy consumption of the proposed ACO-PT and contending algorithms (ACO-FL, RACO, and AODV-ACO) with a maximum of 200 vehicles, (**b**) energy consumption of the proposed ACO-PT and contending algorithms (ACO-FL, RACO, and AODV-ACO) with a maximum of 400 vehicles.

The amount of energy used to perform a specific task is known as energy consumption. Algorithms that use less energy result in higher throughput and prolong the lifetime of the network. Figure 10a shows that the suggested ACO-PT consumed less energy than competing algorithms. With 200 vehicles, the projected ACO-PT consumed 3.35 joules. 

The competing algorithms, on the other hand, consumed 3.85–4.41 joules with the same number of vehicles. When the number of vehicles was raised to 400, the rate of energy consumption followed the same pattern for the proposed algorithm and competing algorithms ACO-FL, RACO, and AODV-ACO. The suggested ACO-PT used 6.15 joules, as depicted in Figure 10b, whereas the competing algorithms used 7.52–8.63 joules. This difference indicates that the proposed algorithm continued to use less energy as the number of vehicles increased.

## 6. Discussion of Results

The proposed ACO-PT heterogeneous algorithm aims to find an efficient and optimal vehicle path by combining the best aspects of two cutting-edge algorithms: pheromone termite and ant-colony optimization. To enhance the reliability of the algorithm, an automatic-vehicle-detection module was added that includes image preprocessing, contour detection, contour matching, and blob detection. Additionally, the proposed algorithm addresses congestion avoidance to improve throughput, energy consumption, and end-to-end latency.

A comparative analysis of the ACO-PT approach with existing state-of-the-art algorithms such as ACO-FL, RACO, and AODV-ACO in terms of throughput, end-to-end delay, and energy consumption was conducted. The results demonstrate that the proposed ACO-PT outperformed other contending methods. The performance and differences are shown in Table 3 and Table 4. The proposed ACO-PT algorithm achieved a throughput of 90.2 kbps with 200 vehicles and 78.2 Kbps with 400 vehicles. Competing methods, on the other hand, achieved a lower throughput between 62.1–68.5 kbps with 200 vehicles and 50–62.1 kbps with 400 vehicles.

Furthermore, the proposed ACO-PT consumed less energy and had a shorter end-to-end latency with a varying number of vehicles. The proposed ACO-PT had an end-to-end latency of 226.3 milliseconds with 200 vehicles and 250 milliseconds with 400 vehicles, as depicted in the tables below. In contrast, the competing methods caused an end-to-end delay of 262.3–363.3 milliseconds with 200 vehicles and 300.8–388.7 milliseconds with 400 vehicles. Hence, with a varying number of vehicles, the suggested ACO-PT used less energy compared to the competing algorithms. In conclusion, the experimental results demonstrate that the proposed ACO-PT algorithm outperforms existing algorithms in terms of throughput, energy consumption, and end-to-end delay. The proposed algorithm is a reliable and efficient solution for finding the optimal path between a source and a destination in urban areas.

## 7. Conclusions and Future Work

This section provides an entire overview of the article, including its results and any weaknesses. The heterogeneous ACO-PT approach is proposed for efficient-route discovery, increasing throughput while decreasing energy consumption and end-to-end latency. The heterogeneous method combines the best aspects of two cutting-edge algorithms (pheromone termite and ant-colony optimization) to produce an efficient and optimum vehicle-pathfinding solution. The pheromone-termite algorithm is based on real-world termite behavior, in which termites utilize pheromones to locate the shortest path to food. The ACO algorithm, on the other hand, is a metaheuristic algorithm that employs the notion of artificial ants to discover the optimum solution to a given issue. It is based on the behavior of actual ants while looking for food.

The first algorithm in our proposed ACO-PT technique, PT, provides an initial path from source to destination, whereas the second, ACO, optimizes the resultant path for accurate and optimal output. Vehicle congestion is a serious problem that has had a significant influence on performance. Congestion avoidance is intended to alleviate this issue by lowering energy usage and throughput. A module for automated vehicle detection is also provided to recognize vehicles at various phases (image preprocessing, contour detection, contour matching, and blob detection).

The experimental findings demonstrate that in terms of throughput, energy consumption, and end-to-end latency, the proposed ACO-PT approach performs better than other approaches, such as ACO-FL, RACO, and AODV-ACO. In comparison to other algorithms, ACO-PT can determine the best path with better throughput, less energy utilization, and shorter end-to-end latency, making it the perfect choice for practical applications.

In the future, we will evaluate the effectiveness of the proposed approach using quality-of-service indicators (minimum arrival time, average arrival time, loss rate, delay variation, and transfer delay). To determine whether this approach is practically appropriate for commercial purposes, the computational complexity will also be determined.

## Figures and Tables

**Table 1:** Contributions of existing techniques.

Works	Heterogeneous Algorithm for Efficient Routing	Features and Strengths	Deficiencies and Vulnerabilities
Zhang et al. [19]	Heterogeneous multidepot cooperative vehicle-routing problem.	Improves vehicle routing by properly selecting transfer points for product transshipment between vehicles of different depots.	Increases cost savings among depots because the grand coalition is not stable in an urban environment.
Khabiri et al. [20]	Energy-aware clustering-based routing in wireless-sensor networks using the cuckoo-optimization algorithm.	Enhances the network lifetime and selects optimal cluster heads through the combination of clustering methods and cuckoo optimization.	The work is restricted to WSNs with stationary sensor nodes. In addition, it increases latency and delay.
Fatemidokht et al. [21]	An improved foundation for the ACO protocol based on fuzzy logic for VANETs.	Testing the effectiveness and performance on NS-2, this algorithm outperforms many algorithms with a higher data-packet delivery ratio and a lower end-to-end delay. It also guarantees road-safety service quality and fulfills some QoS requirements.	This protocol is vulnerable to a number of security threats.
Anandh et al. [22]	A hierarchical routing design and architecture in which nodes in the outer level forward data to inner-level nodes.	Increases energy saving, increases network lifespan, and reduces congestion.	With the use of ACO, certain cluster-head nodes become overburdened with data forwarding.
Oh et al. [23]	A framework that predicts pheromone values using quantum mechanics.	Generates the shortest and most optimal path faster than multiple algorithms.	Increases computation time.
Sindhwani et al. [24]	AODV combined with ACO for data transmission.	Generates a path with the shortest distance, leads to improved throughput, and reduces lost and delayed packets.	Consumes large amounts of network bandwidth and increases network congestion.
Martinez et al. [25]	Estimates statistical variables using a one-way road-network model.	Employs a route-planning algorithm for periodically constrained search regions that generate virtual borders with a lower confidence level.	The search process is reliable but utilizes more energy consumption for optimal path detection.
Haitao et al. [26]	Plans an efficient path from the present to the future.	Proposes the shortest route for packet reduction, throughput increase, and latency reduction.	Addresses only congestion.
Zambrano et al. [27]	Time-dependent vehicle-routing problem with time windows for congestion avoidance.	The proposed method is used for time-dependent vehicle speeds, travel times, vehicle capacities, customer demand, wait times, time windows, servicing times, the effects of dynamic loads, and the impact of travel speeds on vehicle carbon emissions.	The time window is limited to the predicted time. Therefore, the prediction cannot be accurate in most cases.
Yaqoob et al. [28]	Fog-assisted congestion-avoidance method for IoV.	The proposed EEMD makes use of the benefits of fog computing to save communication costs and manage services. The status of each vehicle is frequently updated to a fog server, either directly or via intermediary nodes.	Limited to communication-cost savings but not congestion avoidance.
Liu et al. [29]	An enhanced RES-YOLO detection technique for automated vehicle detection.	Proposing enhanced RES-YOLO for automated vehicle detection employing the capacity to decompose and providing high resilience for non-detection-category data.	No proper proof to demonstrate the automatic-vehicle-detection process.
Our Proposed Method	A novel heterogeneous algorithm called ant-colony optimization with pheromone termite.	The ACO-PT algorithm seeks to provide the shortest effective path from a source point to a destination point to help drivers avoid congested roads in urban areas.	Requires the computational complexity to be determined.

**Table 2:** Summarized simulation parameters and description.

Name of Parameter	Description
Simulator	NS-3.37 simulator
Maximum number of vehicles	400
Transmission range	45 m
Initial energy of the sensor node attached to vehicle	5 Joules
Bandwidth of sensor node	100 Kb/s
Size of simulated network	900 × 900 square meters
Pause time	20 s
Simulation time	10 min
Data-packet size	512 bytes
Data frame	256 bytes
Power consumption	18 mW
Power intensity	−18–15 dBm
MAC protocol	BN-MAC
Tested algorithm	ACO-PT
Competing algorithms	ACO-FL, RACO and AODV-ACO
Mobility model	Manhattan mobility model
Mobility	0–10 m/s

**Table 3:** Comparative analysis of the proposed model with various state-of-the-art algorithms with a maximum of 200 vehicles.

Algorithm	Average Throughput (Kbps)	End-to-End Delay (Milliseconds)	Energy Consumption (Joules)
ACO-FL	An improved foundation for the ACO protocol based on fuzzy logic.	68.5	363.3
RACO	Routing using ant-colony optimization (RACO).	65.2	262.3
AODV-ACO	AODV combined with ACO (AODV-ACO) for data transmission.	62.1	303.3
ACO-PT	A novel heterogeneous algorithm called ant-colony optimization with pheromone termite.	90.2	226.3

**Table 4:** Comparative analysis of the proposed model with various state-of-the-art algorithms with a maximum of 400 vehicles.

Algorithm	Average Throughput (Kbps)	End-to-End Delay (Milliseconds)	Energy Consumption (Joules)
ACO-FL	An improved foundation for the ACO protocol based on fuzzy logic.	62.1	388.7
RACO	Routing using ant-colony optimization (RACO).	56	330.8
AODV-ACO	AODV combined with ACO (AODV-ACO) for data transmission.	50	300.8
ACO-PT	A novel heterogeneous algorithm called ant-colony optimization with pheromone termite.	78.2	250

## Data Availability

Not applicable.
